# Food security is related to adult type 2 diabetes control over time in a United States safety net primary care clinic population

**DOI:** 10.1038/nutd.2017.18

**Published:** 2017-05-15

**Authors:** M U Shalowitz, J S Eng, C O McKinney, J Krohn, B Lapin, C-H Wang, E Nodine

**Affiliations:** 1 NorthShore University HealthSystem Research Institute, Evanston, IL, USA; 2 Department of Pediatrics, University of Chicago, Pritzker School of Medicine, Chicago, IL, USA; 3 Be Well Lake County Diabetes Program, Lake County Health Department and Community Health Center, Waukegan, IL, USA

## Abstract

**Background/Objectives:**

Successful Type 2 diabetes management requires adopting a high nutrient-density diet made up of food items that both meet dietary needs and preferences and can be feasibly obtained on a regular basis. However, access to affordable, nutrient-dense foods often is lacking in poorer neighbourhoods. Therefore, low food security should directly impair glucose control, even when patients have full access to and utilize comprehensive medical management. The present study sought to determine whether food security is related longitudinally to glucose control, over-and-above ongoing medication management, among Type 2 diabetes patients receiving comprehensive care at a Midwestern multi-site federally qualified health centre (FQHC).

**Subjects/Methods:**

In this longitudinal observational study, we completed a baseline assessment of patients’ food security (using the US Household Food Security Module), demographics (via Census items), and diabetes history/management (using a structured clinical encounter form) when patients began receiving diabetes care at the health centre. We then recorded those patients’ A1C levels several times during a 24-month follow-up period. Three hundred and ninety-nine patients (56% with low food security) had a baseline A1c measurement; a subsample of 336 (median age=52 years; 56% female; 60% Hispanic, 27% African American, and 9% White) also had at least one follow-up A1c measurement.

**Results:**

Patients with lower (vs higher) food security were more likely to be on insulin and have higher A1c levels at baseline. Moreover, the disparity in glucose control by food security status persisted throughout the next 2 years.

**Conclusions:**

Although results were based on one multi-site FQHC, potentially limiting their generalizability, they seem to suggest that among Type 2 diabetes patients, low food security directly impairs glucose control—even when patients receive full access to comprehensive medical management—thereby increasing their long-term risks of high morbidity, early mortality, and high health-care utilization and cost.

## Introduction

The growing scourge of Type 2 diabetes is currently one of the most pressing public health concerns facing the United States. In 2012, for example, the disease affected 9.3% of the overall US population (29.1 million people, up from 25.8 million people in 2010), with a disproportionately large impact on the poor and underserved, and was designated the seventh leading cause of death.^1^ To effectively combat this mounting health crisis, it is paramount to understand the factors that facilitate or hamper successful Type 2 diabetes control in everyday life. One likely factor is *low food security*, which is defined by the World Health Organization as lacking physical and/or economic access to ‘sufficient, safe, nutritious food to maintain a healthy and active life’,^2^ and which, similar to Type 2 diabetes, affects 12.7% of US households, especially the poorest and most underserved segments of the population.^3^

Successful Type 2 diabetes control involves a substantial commitment to lifestyle management, including adopting a diet with high nutrient density (in practice, the easy availability of lower caloric density foods and good access to fruits and vegetables) that is both obtainable and meets dietary preferences, in order to prevent or delay the complications from the disease. However, access to affordable, nutrient-dense foods is often lacking in neighbourhoods where a majority of the residents are poor, which is a contributing factor to lower levels of food security among poor individuals. By extension, we expect that these inadequate food resources, due to both financial and geographic constraints, directly impact a patient’s ability to choose nutrient-dense foods necessary for proper glucose control.

Although the proposed relationship between food security and Type 2 diabetes control over time has intuitive appeal and face validity, nearly all prior work examining this link has been limited by use of cross-sectional samples and/or self-reported diabetes diagnosis. For instance, in a study of 40 low-income adults, those with less food security reported lower diabetes self-efficacy, lower adherence to blood glucose monitoring (RR=3.5, *P*=0.008) and more hypoglycemia-related emergency department visits (RR=2.2, *P*=0.007), accompanied by a trend towards higher mean A1c (77 mmol/mol vs 61 mmol/mol (9.2 vs 7.7%), *P*=0.08).^4^ Using NHANES data, a self-reported diagnosis of Type 2 diabetes was associated with low self-reported food security.^5, 6^ A Canadian study^7^ showed that food insecurity was more prevalent among individuals with Type 2 diabetes (9.3 vs 6.8%, *P* <0.05), was not associated with diabetes management services, but was associated with physical inactivity (OR 1.54 [95% CI 1.10−2.17]), lower fruit and vegetable consumption (OR 0.52 [0.33−0.81]), current smoking (OR 1.71 [1.09−2.69]), unmet health-care needs (OR 2.71 [1.74−4.23]), having been an overnight hospital patient (OR 2.08 [1.43−3.04]), and having a mood disorder (OR 2.18 [1.54−3.08]). Also in Canada, decrements in health (Type 2 diabetes)-related quality of life were linked to less food security (−0.07; 95% CI=−0.10, −0.04).^8^ In contrast, a poor, rural, community-based Appalachian sample had low food security, but food security had no relationship to Type 2 diabetes control.^9^

Two more recent projects improved upon past cross-sectional research by examining the relationship between food security and Type 2 diabetes control within pre-/post-test designs, thereby providing an important first step towards elucidating the causal ordering of these processes. Nonetheless, this newer research also suffered from design limitations, and produced mixed results: one study found that food security was not related to A1c at either baseline or the 2-year follow-up,^10^ whereas the other study found that lower food security was related to higher A1c at baseline but predicted more pronounced improvement in A1c when levels were re-measured at the end of the study period.^11^

In the present study, we extend the growing literature on food security and Type 2 diabetes control by testing whether household food security status is related longitudinally to glucose control, over and above ongoing medication management, among patients with Type 2 diabetes served by a Midwestern community health centre. Data were drawn from a structured clinical documentation form (SCDF) for Type 2 diabetes, including a subset of questions from the US Department of Agriculture (USDA) household food insecurity questionnaire, and collected during the course of usual care. Hemoglobin A1C levels were then assessed at several time points over 24 months.

Although this study reports the experience of a specific Midwestern, multi-site federally qualified health centre (FQHC) with a comprehensive diabetes outpatient programme, we extend to prior research by addressing several key design limitations of past studies: we measure diabetes status using an SCDF rather than self-report; we examine a largely non-White, minority patient population, including large groups of Latinos and Blacks; we control for a wider variety of correlates of glucose control; and in contrast to prior prospective studies on this topic, our participants all have multiple A1c measurements over time, allowing us to test whether change in glucose control follows a more complex pattern than simple linear increase or decrease. We hypothesized that patients with Type 2 diabetes who received primary care from this safety net provider would have worse glucose control when they were less food secure, controlling for insulin use and other known correlates of control. We further hypothesized that this maladaptive link would persist over time, even when barriers to comprehensive diabetes management were minimized.

## Materials and methods

### Site

In 2009, a four-hospital private health system in Illinois launched a signature community benefit programme to target care for medically underserved adults with Type 2 diabetes in one of the counties in its service area (County). The programme built on a pre-existing academic-community partnership between the health system and the County Health Department’s community health centre (CHC), and is funded both by the health system and hospital philanthropy. Health system subspecialty physicians contribute care in-kind. Patients were referred for Type 2 diabetes management, mostly by their primary care physicians at the health centre. CHC primary care visits include medication and testing supply assistance, diabetes self-management education, one-on-one and group support, retinal screenings, and fitness programmes. Over time, some patients were referred back to their primary care physicians because they were in good control, others were lost to follow up in the clinical system, although the programme had better retention than the CHC overall. The health system evaluation team and the CHC clinical team collaboratively designed an SCDF geared to clinic flow and Health Resources and Services Administration criteria for diabetes quality care, including quarterly visits and A1c lab testing.^12^ Data are entered into Microsoft ACCESS and downloaded quarterly for analysis, primarily to track the programme performance and to identify barriers and facilitators to care and control.

### Materials

In June 2010, the programme began to assess food security verbally, in English or in Spanish, using the short form of the 18-item US Household Food Security Module from the USDA in USDA Economic Research Service.^13^ The questionnaire was piloted for clinical purposes by the staff due to concerns that the CHC largely serves a ‘food desert’ community, that is, ‘an area in the United States with limited access to affordable and nutritious food, particularly such an area composed of predominantly lower income neighborhoods and communities’.^14^ The questionnaire is included in the programme SCDF and administered at initial and annual visits. It consists of six questions that reflect household food security in the prior 12 months, and scoring is based on the number of ‘positive answers’ to the following questions: (1) The food I bought did not last and I did not have enough money to get more. (2) I did not have enough money to buy healthy meals. (3) You eat less than you thought you should have because you could not afford to buy enough food. (4) Were you hungry but did not eat because you did not have enough money for food? (5) Did you eat less than usual at meals or skip meals because you did not have enough money for food? (If yes to #5) (6) How often did you eat less than usual at meals or skip meals because you did not have enough money for food? The sum of affirmative responses to the six questions in the module is the household’s raw score on the scale, and food security status by raw score is determined as follows: 0–1=food secure, 2–6=food insecure (2–4=low food security, 5–6=very low food security). Data analysed for this report comprise all data from June 2010 when the food security questionnaire was added through November 2013.

Patient demographics, diabetes history and diabetes management questions were drawn from the SCDF. Race-ethnicity information was based on Census questions, as required for federal reporting by the FQHC. Lab values were performed by a certified clinical laboratory under contract to the FQHC.

### Subjects

The adult Type 2 diabetes programme accepted care for 479 patients beginning in June 2009. About 1 year later, the food security questionnaire was incorporated into the initial visits. Three hundred and ninety-nine had a food security score and an associated A1c measurement at baseline; 336 patients with baseline food security and A1c level had at least one follow-up A1c measurement during the next 24 months, and thus were eligible for longitudinal analyses. Patients included vs those excluded in longitudinal analyses were less likely to smoke at baseline (16 vs 27%) and more likely to take metformin (80 vs 67%), along with their other medications.

### Analysis

Based on their responses to the USDA household survey, patients were classified as food secure or insecure. Patients were considered food insecure if their raw survey score exceeded two. Patient characteristics at enrollment were compared between food secure and insecure individuals. Categorical variables were displayed as frequency counts with percentages and were compared using chi-square tests. Continuous variables were shown as means with standard deviations and compared using *t*-tests. Continuous variables whose distributions deviated from normality were displayed as medians with interquartile ranges and compared utilizing Mann−Whitney *U*-tests.

Predictors of A1c levels over the 24-month study period were determined by longitudinal mixed-effects models using maximum likelihood estimating method with unstructured covariance controlling for random effects of individual intercept (random deviation from the mean intercept).^15^ Time was computed as the interval between the baseline (at entry into the comprehensive diabetes programme) A1c measurement date and each follow-up A1c measurement date (two to six measures). Although A1c was expected to be measured quarterly, some patients received more frequent follow-ups while other patients had more irregular follow-up. Time-varying covariates including insulin were tabulated in a similar manner and included in the univariate analysis. A seasonal variable was created based on month of A1c measurement. This population tends to have seasonal employment with varying levels of physical activity. Given A1c measurements reflect the previous 3 months, seasons were defined with a 2-month lag, such that Winter includes A1c’s from February to April, Spring May to July, Summer August to October, and Autumn November to January. Continuous variables were grand-mean centred for inclusion in the model. A fourth degree polynomial growth curve model was used since it fit the data best according to model selection criteria. Covariates with *P*<0.20 in the univariate analysis were candidate variables for multivariable analysis and were manually selected in a backward fashion at 0.05 significance level. All possible interactions were assessed in the final multivariable model. All statistical analyses were conducted in SAS 9.3 (SAS Institute, Cary, NC, USA).

## Results

### Sample and descriptive statistics

Table 1 compares the food secure and food insecure patients by key demographics and medical characteristics. Among the 336 patients included in this study, 44% were food secure and 56% were food insecure (had low or very low food security). By comparison, the average rate of food insecurity in Illinois for 2008–2010 was 12.9%.^15^ The population was 56% female with more females in the food insecurity group than food secure (60 vs 50%). The sample was largely minority in race-ethnicity. Half of the sample was comprised of Spanish-speaking Hispanics (most requiring an interpreter), 10% were English-speaking Hispanics and 27% were African American. There was no difference in food security status. The mean age was 51 years, and the median number of years with diabetes was 5.5. Food insecure patients had significantly longer time since diabetes diagnosis (median years 6.5 vs 5.0, *P*=0.03). Mean body mass index (BMI) was 32.5 kg/m^2^, with 57% of the population obese. Food insecure patients were 61% obese compared to 52% obese in the food secure group (*P*=0.12). Mean A1c was 9.1 mmol/mol in the food insecure group vs 8.56 in the food secure group (*P*=0.04) and significantly fewer patients had <7% A1c levels in the food insecure group as compared to food secure (20 vs 32%, *P*=0.01). Thirty-six percent of food insecure patients were on insulin at enrollment compared to 23% of food secure patients (*P*=0.01). There were no differences in other medications between groups at enrollment. Mean systolic blood pressure was 133.5 and mean diastolic blood pressure was 81.7. These did not differ across food security status.

### Longitudinal analyses

Table 2 shows univariate predictors of A1c levels over 24 months. Less food security was associated with increasing A1c levels over time. Increased age, increased BMI and younger age at diagnosis were associated with decreasing levels of A1c over time. Increasing levels of A1c over time were associated with Hispanic ethnicity, number of years since diabetes diagnosis, and both insulin use at enrollment and insulin use over time. As suspected, Spring, as compared to Summer, was associated with higher A1c levels over time.

The multivariable model is shown in Table 3. A fourth degree polynomial growth curve model was used since it fit the data best according to model section criteria. Less food security remains a significant predictor of increased A1c levels over time (*P*=0.02). Years since diagnosis, Hispanic ethnicity, and increased BMI remain significant predictors of increased A1c levels. Insulin use over time also predicts increasing A1c levels. Three interactions were included and further explored: food security by time, food security by age and years since diagnosis by BMI.

**Figure 1:**
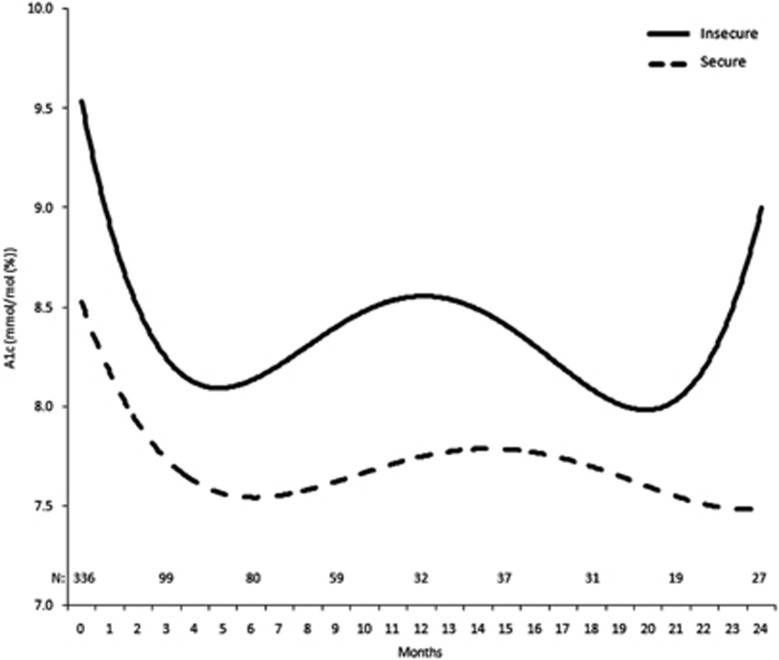
Trajectory of A1c Levels over Time by Initial Food Security Status. The solid line represents A1c levels of food insecure patients, whereas the dashed line represents A1c levels of food secure patients.

**Figure 2:**
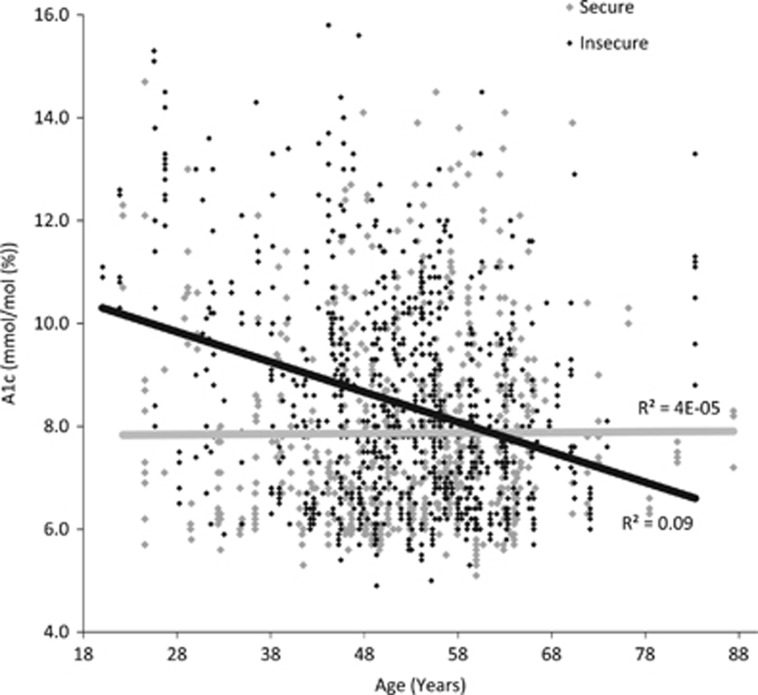
A1c Levels by Age and Food Security Status at Enrollment. The dark grey line represents A1c levels of food insecure patients, whereas the light grey line represents A1c levels of food secure patients.

**Figure 3:**
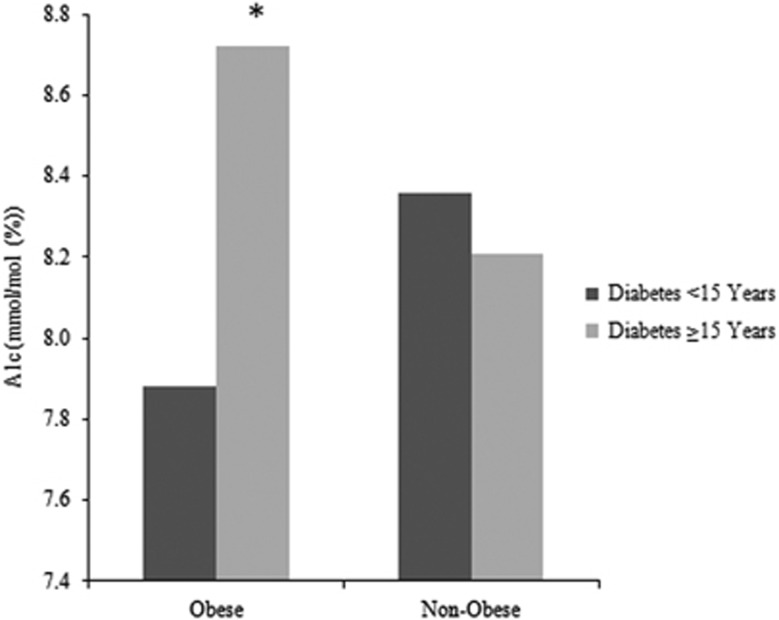
A1c Levels by Years since Diagnosis and Obesity. Dark grey bars represent A1c levels of patients who were diagnosed with diabetes less than 15 years ago, whereas light grey bars represent A1c levels of patients diagnosed with diabetes 15 or more years ago. *P <0.05.

The trajectory of A1c levels over time by food security status is depicted in Figure 1. The food insecure patients show a decrease in A1c levels initially but increase again, decrease, and rise to remain high. The food secure patients decrease and remain fairly stable over the 24-month period. Figure 2 presents a scatter plot of A1c values across age. Food secure patients have stable A1c levels across increasing age, but food insecure patients have significantly higher A1c levels at younger ages with a steady decline in A1c across decades. Figure 3 shows that A1c does not differ by years since diabetes diagnosis in the non-obese patients, but is significantly higher in obese patients with at least 15 years of diabetes.

## Discussion

This paper reports, for the first time, that the perceived adequacy of the food environment is associated with the effectiveness of Type 2 diabetes treatment as measured by glucose control, in a large safety net clinical population of adults with Type 2 diabetes served by a multi-site FQHC in the Midwest. Specifically, controlling for correlates of glucose control, lower levels of food security are directly related to higher A1c. Furthermore, patients who are food secure show consistent benefit from comprehensive diabetes management, but those who are insecure fail to derive this benefit, with the gap in glucose control remaining over time. This finding provides direct support for the importance of the social determinants of diabetes control, which, if replicated, has specific implications for public policy and health-care expenditures over the long run.

These findings are consistent with observations that a diagnosis of diabetes is twice as likely in adults who are food insecure,^16^ and further demonstrate the relationship of food security to the physiology of glucose control, irrespective of obesity status. Obesity is often represented as the common factor associated with both a diagnosis of diabetes and with poverty,^5^ but in our sample,^17^ level of food security was only marginally related to BMI for the entire group, although significant for the subgroup of patients with diabetes longer than 15 years (Figure 3). This finding suggests a largely independent effect of food security on diabetes control, confirmed in our multivariable model.

More recently, two studies have the link between food security and glucose control at two time points only, with mixed results and interpretations. Although we report on a single multisite FQHC, we argue that our findings extend those two studies given the greater diversity in our patient population and the availability of multiple measures of A1c over time. The first study^10^ was a population-based, stratified random sample of Puerto Ricans in Boston who had self-reported their diabetes and food insecurity status at an unspecified point in the study, analysing within-subject change in mean A1c measured first at baseline and at a 2-year follow-up. Contrary to predictions, analyses adjusting for age, sex, education, income-to-poverty ratio, BMI, physical activity, alcohol use, and use of oral glucose-lowering medications and insulin, found that food security status was not significant related to A1c levels, either at the outset of the study or 2 years later.

Findings from a second, more rigorous longitudinal study^11^ more closely replicated cross-sectional findings on food insecurity and diabetes control, but analyses of prospective effects produced counterintuitive results, perhaps because the study was a secondary data analysis of a clinic-clustered, randomized intervention trial that examined diabetes self-management intervention in a sample of mostly White, low-income patients, and was not focused primarily on food security. Nonetheless, food security status was assessed at baseline along with other demographics and diabetes control was measured both at the outset of the study and a short 9-month follow-up. Consistent with prior research, regression models that adjusted for age, sex, race, income and intervention condition, revealed that food insecure individuals had 0.59% higher A1c levels at baseline than did people who were food secure (95% CI=0.19, 0.98). However, although there was significant interaction of food insecurity with time, it had an unexpected pattern: food secure patients (−0.01% 95% CI=−0.19, 0.16) showed no change in A1c from baseline to follow-up, whereas A1c significantly decreased among patients who were food insecure (−0.38; 95% CI=−0.69, −0.08), suggesting less food security led to larger improvement in diabetes control over this short period of time.

Plausible explanations for the relationship we found between glucose control and level of food security are both practical and theoretical. Given that diabetes requires a considerable investment in lifestyle change, poor access to foods of high nutrient density in the community makes actualizing dietary management more difficult, if not impossible, regardless of knowledge and commitment to change. Further, financial assistance programmes and low or hourly wage jobs may cause families to experience resource cycling paycheck-to-paycheck. Resource cycling may have potential emotional, material and physiological influences on diabetes control. Fluctuations in available cash could result in cycling in the purchase of food of good nutrient density, as well as of medication, and consequently produce swings in glucose control. In practice, this is a particular problem when patients are treated with sulfonylureas. This class of diabetes medications is available in a generic form, so they are used by preference with poorer patients before trying more expensive classes of treatment. However, this class of medications, more than other classes of oral medication, causes hypoglycemia when a patient skips meals. Erratic glucose levels that patients experience when they fast or skip meals also undermine compliance to the treatment regimen because of the unpleasant side effects of hypoglycemia (and hyperglycemia if they compensate by overeating later). For example, during Ramadan, clinicians may alter observant Muslim patients’ medication lists during the fasting period, hopefully helping to avoid episodes of hypoglycemia (clinical note from Nodine). However, this change interferes with maintaining their A1c goals. Finally, these fluctuations are sources of stress to which the patient must continually adapt, and thus have both a physiological and psychological cost. In fact, recent evidence suggests an association between food insecurity and elevation of the pro-inflammatory cytokine C-reactive protein.^18^ This newly demonstrated association elevates the risk for cardiovascular disease for diabetes patients who are overweight.

There are several limitations to this study. First, these data were drawn from a specific Midwestern multi-site FQHC and we do not know the rates of food security in its larger FQHC population. Therefore, we cannot make statements about whether food security levels among the Type 2 diabetes programme participants differ from those in the overall clinic population. Similarly, we do not know whether patients with diabetes who were not referred to the diabetes programme (who are cared for in the general medical or family practice clinics) show the same relationship between glucose control and food security. In addition, the USDA food security screen relies on patient self-report. This study does not verify actual food availability among these programme participants. While these limitations are threats to generalizability, we argue that they do not alter the main message of this paper.

In sum, our findings suggest that patients with Type 2 diabetes mellitus with lower food security have worse glucose control than those who are food secure, placing food insecure patients at higher risk for long-term higher morbidity, early mortality, and high health-care utilization and cost. Furthermore, even when provided with full access to comprehensive diabetes management, in the first 24 months of observation, patients with low food security do not derive the same benefit as those who are food secure.

Future studies should replicate these findings in other locations and evaluate whether the effect of food insecurity demonstrated in the present study holds for other measures of diabetes control and quality of care. Moreover, future research should examine predictive models for diabetes control using a broader range of social and biological determinants. This paper’s demonstration of the link between social and biological determinants for two urgent public health emergencies underscores the need for a more comprehensive, community-based approach to care. Improving health and quality of life for these patients with diabetes will demand a multi-sector approach combining medicine, public health, the built environment and public policy.

## Figures and Tables

**Table 1:** Patient characteristics at enrollment by initial food security status

*Characteristics*	*Total* N *(%)*	*Food secure* N *(%)*	*Food insecure* N *(%)*	P*-value*^a^
Total number of patients	*336*	*149 (44.4)*	*187 (55.7)*	
Current age (years) − Mean±s.d.	51.8±10.9	52.1±11.5	51.5±10.5	0.579
Female gender	188 (56.0)	75 (50.3)	113 (60.4)	0.064
BMI (kg/m^2^) − Mean±s.d.	32.5±7.5	31.8±6.5	33.1±8.1	0.104
Obese (BMI≥30)	186 (57.2)	76 (52.4)	110 (61.1)	0.115
				
*Race, ethnicity and language*				
African-American non-Hispanic	90 (26.8)	37 (24.8)	53 (28.3)	0.346
White non-Hispanic	29 (8.6)	9 (6.0)	20 (10.7)	
Hispanic-Spanish	167 (49.7)	76 (51.0)	91 (48.7)	
Hispanic-English	32 (9.5)	17 (11.4)	15 (8.0)	
Other	18 (5.4)	10 (6.7)	8 (4.3)	
Age at diagnosis (years) − Mean±s.d.	44.3±11.1	45.4±11.9	43.4±10.5	0.119
Years since diagnosis − Median (Q1, Q3)	5.5 (1.0, 11.0)	5.0 (1.0, 10.0)	6.5 (1.0, 12.0)	*0.031*
Tobacco use	48 (14.3)	15 (10.1)	33 (17.7)	0.049
Systolic blood pressure − Mean±s.d.	133.5±19.9	133.8±19.4	133.2±20.4	0.807
Diastolic blood pressure − Mean±s.d.	81.7±11.9	82.4±12.3	81.2±11.5	0.392
Physical activity	227 (67.6)	100 (67.1)	127 (67.9)	0.876
Sum hours per week − Median (Q1, Q3)	3.0 (2.2, 7.0)	2.5 (2.0, 7.0)	3.0 (2.5, 7.0)	0.304
HgbA1c − Mean±s.d.	8.87±2.37	8.56±2.35	9.11±2.37	*0.036*
<7%	85 (25.3)	48 (32.2)	37 (19.8)	*0.009*
LDL − Mean±s.d.	102.4±35.8	99.4±32.9	104.8±37.9	0.207
Microalbumin − Median (Q1, Q3)	11.0 (5.0, 42.0)	9.0 (5.0, 38.0)	12.5 (5.0, 43.0)	0.158
Metformin	272 (81.0)	123 (82.6)	149 (79.7)	0.506
Statins	165 (49.1)	75 (50.3)	90 (48.1)	0.688
Insulin	101 (30.1)	34 (22.8)	67 (35.8)	*0.010*
Number of A1c measurements during the study period – Median (Q1, Q3)	4.0 (2.0, 6.0)	4.0 (2.0, 6.0)	4.0 (2.0, 6.0)	0.607

a Italicized *P*-values are statistically significant at *P*<0.05.

**Table 2:** Predictors of A1c levels over time (*P*<0.20)

*Predictors*	*Estimate*	*SE*	P*-value*^a^
Age at enrollment (years)	−0.028	0.008	*<0.001*
Female gender	−0.305	0.183	0.096
BMI at enrollment (kg/m^2^)	−0.039	0.012	*0.001*
Obese	−0.575	0.186	*0.002*
			
*Race, ethnicity and language*			
White non-Hispanic	Reference		
African-American non-Hispanic	0.349	0.349	0.317
Hispanic-Spanish	0.704	0.328	*0.032*
Hispanic-English	1.135	0.420	*0.007*
Other	0.569	0.496	0.252
Age at diagnosis (years)	−0.045	0.008	*<0.001*
Years since diagnosis	0.038	0.012	*0.002*
Physical activity level at enrollment	−0.330	0.194	0.090
LDL at enrollment	0.004	0.003	0.117
Insulin at enrollment	1.299	0.187	*<0.001*
Time-varying insulin	0.814	0.125	*<0.001*
Food insecure (vs secure) at enrollment	0.452	0.182	*0.013*
			
*Season of A1c measurement*^a^			
Summer	Reference		
Autumn	0.022	0.110	0.842
Winter	0.124	0.103	0.227
Spring	0.282	0.106	*0.008*
Number of A1c measurements	−0.098	0.058	0.095

a Italicized *P*-values are statistically significant at *P*<0.05.

b Since A1c measures reflect the previous 3 months, seasons are lagged by 2 months (that is, Winter includes February, March and April).

**Table 3:** Multivariable model for predictors of A1c levels over time

*Predictors*	*Estimate*	*SE*	P*-value*^a^
Months (linear)	−0.472	0.061	*<0.001*
Months (square)	0.075	0.013	*<0.001*
Months (cubic)	−0.004	0.001	*<0.001*
Months (quadratic)	0.0001	0.00002	*<0.001*
Food insecure at enrollment	0.428	0.178	*0.017*
Food insecure × months (linear)	−0.023	0.011	*0.036*
Age at enrollment	−0.006	0.011	0.571
Food insecure × age	−0.053	0.014	*<0.001*
BMI at enrollment	−0.064	0.016	*<0.001*
Years since diagnosis	0.038	0.012	*0.002*
Years since diagnosis × BMI	0.004	0.001	*0.006*
			
*Race, ethnicity and language*			
White non-Hispanic	Reference		
African-American non-Hispanic	0.426	0.298	0.153
Hispanic-Spanish	0.563	0.286	*0.049*
Hispanic-English	1.010	0.368	*0.006*
Other	0.693	0.426	0.104
Time-varying insulin	0.705	0.127	*<0.001*

a Italicized *P*-values are statistically significant at *P*<0.05.
